# Effects of treadmill with different intensities on bone quality and muscle properties in adult rats

**DOI:** 10.1186/s12938-019-0728-0

**Published:** 2019-11-12

**Authors:** Zhehao Liu, Jiazi Gao, He Gong

**Affiliations:** Department of Engineering Mechanics, Jilin University, Changchun, 130022 People’s Republic of China

**Keywords:** Treadmill exercise, Exercise intensity, Femurs, Biomechanical properties, Multiscale analysis

## Abstract

**Background:**

Bone is a dynamically hierarchical material that can be divided into length scales of several orders of magnitude. Exercise can cause bone deformation, which in turn affects bone mass and structure. This study aimed to study the effects of treadmill running with different intensities on the long bone integrity and muscle biomechanical properties of adult male rats.

**Methods:**

Forty-eight 5-month-old male SD rats were randomly divided into 4 groups: i.e., sedentary group (SED), exercise with speed of 12 m/min group (EX12), 16 m/min group (EX16), and 20 m/min group (EX20). The exercise was carried out for 30 min every day, 5 days a week for 4 weeks. The femurs were examined using three-point bending test, microcomputer tomography scanning and nanoindentation test; the soleus muscle was dissected for tensile test; ALP and TRACP concentrations were measured by serum analysis.

**Results:**

The failure load was significantly increased by the EX12 group, whereas the elastic modulus was not significantly changed. The microstructure and mineral densities of the trabecular and cortical bone were significantly improved by the EX12 group. The mechanical properties of the soleus muscle were significantly increased by treadmill exercise. Bone formation showed significant increase by the EX12 group. Statistically higher nanomechanical properties of cortical bone were detected in the EX12 group.

**Conclusion:**

The speed of 12 m/min resulted in significant changes in the microstructure and biomechanical properties of bone; besides, it significantly increased the ultimate load of the soleus muscle. The different intensities of treadmill running in this study provide an experimental basis for the selection of exercise intensity for adult male rats.

## Background

Osteoporosis is a type of bone disease characterized by decrease in bone quality and deterioration of bone microstructure, which leads to an increase in bone fragility and susceptibility to fractures [1, 2]. More than 200 million people suffer from osteoporosis worldwide [3]. As a major public health problem all over the world, osteoporosis imposes severe health threats and financial burdens on the patients and their families.

Currently, a wide variety of drugs, such as alendronate sodium, raloxifene, and teriparatide, are used for the treatment of osteoporosis. However, these drugs are associated with serious adverse reactions, e.g., high blood pressure, hectic fever, and thrombus formation [4]. Alternatively, non-drug therapy has become a popular method for treating osteoporosis due to its low price, applicability, and good treatment outcomes. According to the previous studies, exercises such as jumping, jogging, and swimming can improve bone quality, density, and strength [5–7]. Physical exercise, in particular treadmill running, has been widely used in animal models associated with osteoporosis, as its mechanism is basically similar to human running on a treadmill [8, 9].

The data obtained from animal experiments do not fully represent the condition of the human body. However, animal experiments can replicate the development of the disease and can be studied for specific situations, and can be combined with a variety of experimental methods to further study the mechanism of disease development. In addition, animal experiments can explore the effectiveness of different therapeutic options and provide a theoretical basis for their clinical application. Due to the similarities of bone morphology and structure between rat and human, rats have become an excellent animal model for studying bone diseases. Rats are also the most commonly used experimental animals for studying the effects of exercise on the musculoskeletal system. Compared to female rats, male rats can reduce estrogen intervention and are more suitable for treadmill running.

Bone strength is related to not only its structure and geometry, but also the external load and the bone material properties [10, 11]. Studies have demonstrated that there is a “feedback system” in bone tissue, which is able to sense strain changes inside the bone [12]. Furthermore, In addition to age, gender, nutritional status and energy intake, the strain environment around the bone is also an important factor affecting bone mass, which decreased in the position where strain is reduced and added in the position where strain is increased, affecting the size, shape, and structure of the bone. Bone has a multi-scale hierarchical structure at macroscopic, microscopic, and nanoscopic levels with various characteristics at different scales [13]. Thus, studies at different scales are required to better understand the relationship between bone strength and structure.

Since the mechanical stimulus generated by treadmill running is mostly generated by impact with the ground or muscle contraction, it is essential to investigate the muscle force. The soleus and the gastrocnemius muscles are both posterior muscles of the lower limbs, and belong to the antigravity muscles that maintain body posture or exercise against gravity. Moreover, the soleus and gastrocnemius muscles are dominated by slow twitch (ST) and fast twitch (FT) fibers, respectively [14]. Contraction of the slow twitch fibers is dominant during endurance training; while the fast twitch fibers also participate in contraction during prolonged exercises. Therefore, treadmill running affects the soleus muscle first, and then the gastrocnemius muscle is affected. There are many reports about the effects of exercise on the soleus muscle under micro-gravity [15–18], which revealed that treadmill running could alleviate muscle atrophy to a certain extent in astronauts caused by long-term exposure to micro-gravity. Moreover, high-intensity treadmill running led to 59% less muscle loss in astronauts compared to low-intensity exercise [18]. As one of the main muscles providing calf tension and directly participating in the eccentric contraction, the soleus muscle has important significance in terms of its biomechanical properties.

A recent study of a 1-month-old female rat backpack with weight-bearing and moderate intensity treadmill found that when rats ran at moderate intensity with an additional 12% weight-bearing level, bone formation was promoted and cancellous bone microstructure was significantly improved [19]. The choice of age and gender has an important impact on the experimental results. Previous study showed that treadmill running had different adaptability to bone structure, biomechanical properties and molecular signals between 12-week-old male and female rats [20]. Compared to female rats, male rats reduced estrogen intervention and were more suitable for treadmill running. Adult male rat reaches peak bone mass, and it can reduce the effect of age on bone mass in the experiment. Therefore, 5-month-old male rats were used to study the effects of different intensities treadmill running on bone mass and muscle biomechanical properties in adult male rats. The study of bones and muscles can provide a more comprehensive experimental basis for the selection of exercise intensity in adult male rats.

In this study, treadmill running at different intensity levels was carried out, in which multi-level test and analyses, including serum analysis, mechanical tensile test of soleus muscle, three-point bending test, micro-CT scanning, and nanoindentation test, were performed to investigate the effects of different levels of mechanical stimuli on the long bone quality and muscle performance of adult male rats, thereby providing feasible suggestions for selecting a proper exercise intensity for adult males.

## Results

### Mechanical properties and weight of lower soleus muscle

**Fig. 1:**
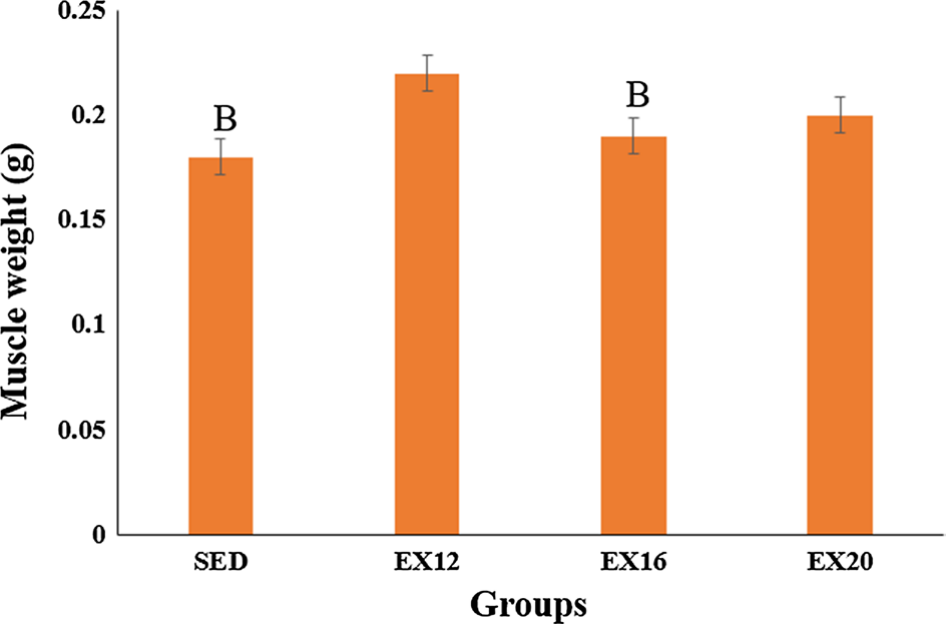
Soleus muscle weight. The error line represents SD. ^B^ Statistically different from EX12 group (*p* < 0.05)

The soleus muscle is one of the most important lower limb muscles. Table 1 shows the ultimate load and ultimate displacement values obtained from the soleus muscle tensile tests. It can be seen that the ultimate load and ultimate displacement values of the exercise groups were significantly higher than those of the SED group (*p* < 0.05). Although there were no significant differences between the EX12 group and the other exercise groups, the EX12 group had the maximum ultimate load and the minimum ultimate displacement. The EX12 group had the maximum soleus muscle weight, which was 18.18% and 13.64% greater than those of the SED group and the EX16 group, respectively (*p *< 0.05) (Fig. 1).

**Table 1:** The ultimate load and ultimate displacement values of soleus muscle obtained from tensile test, mean ± SD

Groups	Ultimate load (N)	Ultimate displacement (mm)
SED	2.82 ± 0.38	7.75 ± 1.70
EX12	3.51 ± 0.55^a^	9.45 ± 2.13^a^
EX16	3.36 ± 0.46^a^	9.77 ± 1.77^a^
EX20	3.33 ± 0.53^a^	9.92 ± 2.10^a^

*SED* sedentary control group; *EX12* exercise group with a speed of 12 m/min; *EX16* exercise group with a speed of 16 m/min; *EX20* exercise group with a speed of 20 m/min

^a^Statistically different from SED group (*p* < 0.05)

### Activity evaluation of osteoblasts and osteoclasts by the serum analysis

**Fig. 2:**
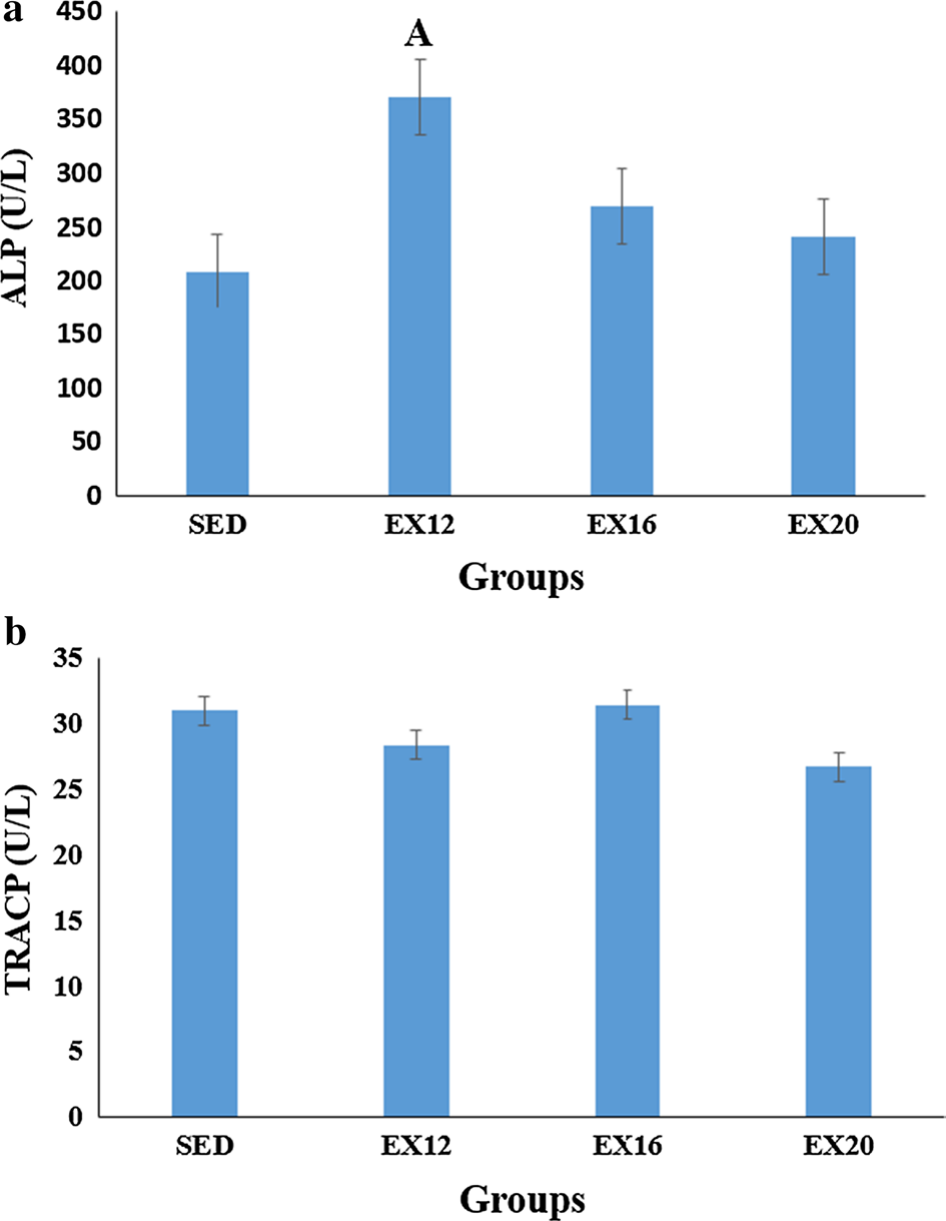
The concentrations of ALP and TRACP obtained from serum analysis. The error line represents SD. **a** ALP Alkaline phosphatase. **b** TRACP Tartrate-resistant acid phosphatase. ^A^ Statistically different from SED group (*p* < 0.05)

The concentrations of alkaline phosphatase (ALP) and tartrate-resistant acid phosphatase (TRACP) obtained by serum analysis are shown in Fig. 2. As can be seen from Fig. 2a, higher ALP concentrations were found in the exercise groups compared with the SED group, and the EX12 group had statistically higher ALP concentration than the SED group (*p *< 0.05). No statistical difference was found in TRACP concentration for all groups as shown in Fig. 2b; however, TRACP concentration in the EX12 and EX20 groups were smaller than that in the SED group (*p *> 0.05), while TRACP concentration in the EX16 group was similar with the SED group.

### The failure load and elastic modulus of the femur evaluated by the three-point bending mechanical test

The failure load and elastic modulus values of the femurs were obtained by the three-point bending mechanical test as shown in Table 2. It was found that the failure loads of the SED and EX20 groups were significantly different from that of the EX12 group (*p *< 0.05). The EX12 group had the maximum failure load and the EX20 group had the minimum failure load. No statistical difference in the elastic modulus was found for all the groups (*p *> 0.05). The elastic modulus in the EX16 groups was smaller than those in the EX12 and EX20 groups (*p *> 0.05). On the one hand, it may be related to the porosity and the *ET/HT* ratio. The *E/H* ratio describes the material deformation during the indentation process, which is a crucial indicator for the toughness of materials [25]. On the other hand, it may be related to the chemical composition of the cortical one and the arrangement of the collagen fibers [26, 27].

**Table 2:** The failure load and elastic modulus values of femurs obtained from three-point bending mechanical test, mean ± SD

Groups	Failure load (N)	Elastic modulus (GPa)
SED	124.52 ± 30.50	6.85 ± 2.72
EX12	155.62 ± 20.77^a,b^	7.39 ± 1.32
EX16	142.21 ± 33.71	7.24 ± 1.95
EX20	122.49 ± 24.17	8.00 ± 2.76

*SED* sedentary control group; *EX12* exercise group with a speed of 12 m/min; *EX16* exercise group with a speed of 16 m/min; *EX20* exercise group with a speed of 20 m/min

^a^Statistically different from SED group (*p *< 0.05)

^b^Statistically different from EX20 group (*p *< 0.05)

### Microarchitectural evaluation of the left femur by micro-CT scanning

The 3D microstructure parameters of femoral distal trabecular bone were obtained by CTAn software as shown in Table 3. The EX12 group had the maximum bone volume fraction (BV/TV), trabecular bone mineral density (Tb.BMD), trabecular thickness (Tb.Th), trabecular number (Tb.N), structure model index (SMI), while the trabecular separation (Tb.Sp) was the minimum; BV/TV, Tb.BMD and Tb.Th in the EX12 group were statistically higher than those in the SED, EX16 and EX20 groups (*p *< 0.05); significantly lower Tb.Sp was found in the EX16 group compared with the SED group (*p *< 0.05); The EX12 group had the maximum structure model index (SMI) among all the components. Slightly lower SMI was found in the EX16 and EX20 groups than that of the SED group. SMI in the EX16 group was significantly lower than that in the EX12 group (*p *< 0.05).

**Table 3:** Microarchitecture parameters of trabecular bone in the distal femur evaluated by micro-CT, mean ± SD

Groups	BV/TV (%)	Tb.BMD (g/cm^3^)	Tb.Th (mm)	Tb.N (1/mm)	Tb.Sp (mm)	SMI
SED	44.70 ± 6.24	0.53 ± 0.06	0.14 ± 0.01	3.32 ± 0.57	0.38 ± 0.10	0.69 ± 0.41
EX12	53.12 ± 8.09^a,c,d^	0.61 ± 0.07^a,c,d^	0.16 ± 0.02^a,c,d^	3.60 ± 0.40	0.30 ± 0.08	1.14 ± 0.73
EX16	45.89 ± 6.98	0.55 ± 0.06	0.14 ± 0.01	3.37 ± 0.50	0.30 ± 0.07^a^	0.60 ± 0.39^b^
EX20	42.09 ± 7.67	0.51 ± 0.06	0.12 ± 0.02^a,c^	3.42 ± 0.52	0.32 ± 0.10	0.58 ± 0.50

*Tb.BMD* trabecular bone mineral density, *BV/TV* bone volume fraction, *Tb.Th* trabecular thickness, *Tb.N* trabecular number, *Tb.Sp* trabecular separation, *SMI* structure model index, *SED* sedentary control group, *EX12* exercise group with a speed of 12 m/min, *EX16* exercise group with a speed of 16 m/min, *EX20* exercise group with a speed of 20 m/min

^a^Statistically different from SED group (*p *< 0.05)

^b^Statistically different from EX12 group (*p *< 0.05)

^c^Statistically different from EX16 group (*p *< 0.05)

^d^Statistically different from EX20 group (*p *< 0.05)

The 3D microstructure parameters of cortical bone in the femoral diaphysis were obtained by CTAn software as shown in Table 4. Statistically lower cortical bone mineral density (Ct.BMD) was found in the SED and EX16 groups compared with the EX12 group (*p *< 0.05). Cortical bone porosity (Ct.P) in the EX12 and SED groups was significantly lower than that in the EX16 group (*p *< 0.05). Significantly higher cortical bone thickness (Ct.Th) was detected in the EX12 group compared with the SED and EX20 groups (*p *< 0.05).

**Table 4:** Microarchitecture parameters of cortical bone in the femur diaphysis evaluated by micro-CT scanning, mean ± SD

Groups	Ct.BMD (g/cm^3^)	Ct.P (%)	Ct.Th (mm)
SED	1.67 ± 0.03	2.51 ± 0.60	32.40 ± 3.32
EX12	1.71 ± 0.03^a,c^	2.25 ± 0.45	35.23 ± 2.97^a,d^
EX16	1.67 ± 0.05	3.17 ± 0.71^a,b^	34.41 ± 4.48
EX20	1.68 ± 0.03	2.67 ± 0.59	32.40 ± 2.50

*Ct.BMD* cortical bone mineral density, *Ct.P* cortical bone porosity, *Ct.Th* cortical bone thickness. *SED* sedentary control group, *EX12* exercise group with a speed of 12 m/min, *EX16* exercise group with a speed of 16 m/min, *EX20* exercise group with a speed of 20 m/min

^a^Statistically different from SED group (*p *< 0.05)

^b^Statistically different from EX12 group (*p *< 0.05)

^c^Statistically different from EX16 group (*p *< 0.05)

^d^Statistically different from EX20 group (*p *< 0.05)

### Elastic modulus and hardness measured by nanoindentation test

Modulus of indentation (*E*), indentation hardness (*H*) and the ratio of indentation modulus to indentation hardness (*E/H*) of cortical bone in the femoral diaphysis were obtained by nanoindentation tests as shown in Table 5. It can be found that statistically greater longitudinal indentation hardness (*HL*), transverse modulus of indentation (*ET*), ratio of transverse indentation modulus to indentation hardness (*ET/HT*) were detected in the EX12 group compared with the SED group; ratio of longitudinal indentation modulus to indentation hardness (*EL/HL*) was statistically smaller in the SED group (*p *< 0.05). No statistical differences were found in longitudinal modulus of indentation (*EL*), *HL* and *EL/HL* ratio between the EX16 and SED groups (*p *> 0.05); *ET* and transverse indentation hardness (*HT*) were significantly greater compared with the SED group (*p *< 0.05). *EL*, *HL*, *HT* and *ET/HT* ratio in the EX20 group were significantly smaller than those in the SED group (*p *< 0.05).

**Table 5:** *E*, *H* and *E/H* ratio of cortical bone obtained by nanoindentation tests, mean ± SD

	SED	EX12	EX16	EX20
Ct.L				
*EL* (GPa)	23.57 ± 3.85	24.15 ± 3.85	25.04 ± 3.84	20.71 ± 3.03^a,b,c^
*HL* (GPa)	1.01 ± 0.21^d^	1.27 ± 0.49^a,c,d^	1.01 ± 0.17	0.92 ± 0.08
*EL/HL*	24.10 ± 4.67^b^	21.62 ± 7.30	25.03 ± 3.26^b^	22.62 ± 3.01
Ct.T				
*ET* (GPa)	19.69 ± 2.97	21.56 ± 2.29^a,d^	20.97 ± 1.83^a,d^	18.74 ± 1.67
*HT* (GPa)	0.88 ± 0.20^c,d^	0.89 ± 0.24^d^	0.97 ± 0.19^d^	0.74 ± 0.08
*ET/HT*	22.87 ± 2.88^b,d^	25.91 ± 6.83	22.40 ± 4.31^b^	22.55 ± 3.31

*n* = 12 in each group

*Ct.L* longitudinal cortical bone, *Ct.T* transverse cortical bone, *EL* longitudinal modulus of indentation, *ET* transverse modulus of indentation, *HL* longitudinal indentation hardness, *HT* transverse indentation hardness, *EL/HL* ratio of longitudinal indentation modulus to indentation hardness, *ET/HT* ratio of transverse indentation modulus to indentation hardness, *SED* sedentary control group, *EX12* exercise group with a speed of 12 m/min, *EX16* exercise group with a speed of 16 m/min, *EX20* exercise group with a speed of 20 m/min

^a^Statistically different from SED (*p *< 0.05)

^b^Statistically different from EX12 (*p *< 0.05)

^c^Statistically different from EX16 (*p *< 0.05)

^d^Statistically different from EX20 group (*p *< 0.05)

## Discussion

In this study, forty-eight 5-month-old male SD rats were divided into three intensity exercise groups and one control group. Macroscopic, microscopic, and nanoscopic perspectives were used to investigate the effects of different treadmill exercise intensities on rat femur. Three-point bending test, micro-CT scanning, and nanoindentation test were performed to study bone quality from multiple perspectives. Moreover, since the mechanical stimulus generated during treadmill exercise is derived not only from gravity, but also from muscle contraction, the mechanical properties of the soleus muscle were also investigated. It was found that exercise at a speed of 12 m/min could significantly increase the long bone quality and increase the ultimate load carried by the soleus muscle of rats.

In recent years, female rats were used in most of the research on the effect of treadmill running on bone mass and strength, which were in the growing period or elderly; while there were relatively few studies on the effect of treadmill running on bone mass in male rats, 4- to 10-week-old growing period [28, 29] or 15- to 23-month-old [30, 31] rats were predominant. From the growth phase to the adult phase and the elderly phase, bone mass changes significantly, so the study for each phase is essential. Previous studies have only observed the effects of treadmill running on bone [6] or muscle [32], and rarely combine together. Adult male rats were used in this study, and the best form of exercise to improve bone quality in adulthood was explored by combining bone and muscle investigations. In addition, previous studies on skeletal muscles in the lower limb were usually focused on the gastrocnemius muscle, and relatively less attention was paid to the soleus muscle. These research protocols focused on the muscle fiber mass [33] and functional characteristics [18]. Studying the mechanical properties of soleus muscle is important for the treatment of muscle atrophy.

Mechanical stimuli play an important role in bone strength [34]. Exercise with different intensity, frequency, and duration produces different mechanical stimuli, which has different effects [8]. In this study, the EX12 group had the maximum failure load, which was significantly greater than those of the SED and the EX20 groups (*p *< 0.05); while the failure load in the EX20 group was smaller than that of the SED group. Bone is a living organ with functional adaptability. It can adjust itself according to the surrounding mechanical environment, developing the optimal structure able to bear the applied loads. Bone can achieve the maximum structural strength with the least materials [35]. Appropriate mechanical stimuli can increase bone quality and improve bone structure; while excessive mechanical stimuli may cause bone tissue deterioration in quality and microstructure, and decrease the mechanical properties [36]. Previous studies on 5-week-old male rats also revealed that, after high-intensity treadmill training, both tibial strength and proximal bone mineral density (BMD) reduced, while the trabeculae in the epiphysis thinned [37]. The treadmill running with 20 m/min speed in this study caused excessive mechanical stimulus to rat femur, which resulted in the decrease in failure load.

The mechanical properties of bone are closely related to its microstructure. Bone strength can be effectively predicted by combining three-point bending test and micro-CT scanning [38, 39]. Compared to the microstructure parameters of the trabecular bone in other groups, the EX12 group demonstrated higher BV/TV, Tb.BMD, and Tb.Th (*p *< 0.05). The EX12 group significantly improved the trabecular structure, increased bone density, and tightened trabecular arrangement as compared to the SED group. These results are consistent with the results from the previous studies, which indicated that exercise can improve the trabecular bone structure in rats [40]. For cortical bone, the EX12 group had higher Ct.BMD and Ct.Th compared to the SED group (*p *< 0.05); while Ct.P was the minimum among all groups. This indicated that applying a mechanical stimulus with a velocity of 12 m/min can increase bone density and cortical bone thickness.

It has been reported in the literature that exercise can increase the levels of growth hormone (GH), prostaglandin E2 (PGE2), parathyroid hormone (PTH), and thyroid hormone (TH) [41]. Among them, PGE2 can stimulate mesenchymal stem cells to differentiate into osteoblasts [42]. ALP is an indicator of bone formation, and is closely related to the activity of osteoblasts. Analysis of ALP in serum can effectively reveal the effect of treadmill running on the active of osteoblasts [43]. Recent studies showed that physical exercise has impacts on the amount and osteogenic differentiation potential of mesenchymal stem cells. After running on the treadmill for 5 weeks, the mesenchymal stem cells of 4-week-old male C57BI/6 mice significantly increased, and the ALP activity also increased [44]. In the present study, no significant difference was found in the serum TRACP concentration among groups. The ALP concentrations in the exercise groups were higher than that in the SED group; while the ALP concentration of the EX12 group was significantly higher than that of the SED group (*p *< 0.05). The results suggested that treadmill exercise with a 12 m/min speed contributed to increasing bone formation, but did not remarkably suppress bone resorption, which was consistent with the increase in bone density in the EX12 group, as observed by micro-CT evaluation. Previous studies have shown different results on whether treadmill running can reduce TRACP concentration. A study on the treadmill running in rats also found that rats increased bone formation after 8 weeks of running without affecting bone resorption [19]. This is consistent with our current conclusion. Other researchers found that treadmill running not only increases bone formation but also inhibits bone resorption [45]. This may be related to different animal species, age and experimental design. In this study, however, the EX16 and SED groups demonstrated similar TRACP concentrations, which should be further investigated.

Nanoindentation is an effective method for measuring nanomechanical properties, and has been widely used in studying the mechanical properties of bone tissue [46]. In the present study, the longitudinal hardness (*HL*) and the transverse elastic modulus (*ET*) of the EX12 group were both significantly improved compared to the SED group (*p *< 0.05), indicating that the nanomechanical properties in the EX12 group were superior to those in other groups. The *E/H* ratio describes the material deformation during the indentation process, which is proportional to the fracture toughness; thus, it is a crucial indicator for the toughness of materials [25]. The *ET/HT* ratio of the EX12 group was significantly greater than that of the SED group (*p *< 0.05), suggesting that the EX12 group had a high transverse fracture resistance. In addition, we also observed that the variation tendencies in elastic modulus and hardness were consistent.

In human activities, most of the loads acting on bones are produced by externally exerted forces and skeletal muscle contraction [47]. In this study, the ultimate load and the ultimate displacement values of the soleus muscle in the exercise groups were significantly higher than those in the SED group (*p *< 0.05). The EX12 group demonstrated the maximum ultimate load and the soleus muscle weight. Mechanical environment is a key factor in maintaining musculoskeletal functions [48]. Exercise increases the muscle myosin and actin contents [49]; and during exercise, muscle contraction increases the number of cells in the muscle tendon, thus improving the load-bearing capacity of the muscle [50]. The increase in the soleus muscle weight in the exercise groups may be due to the apparent “demand” for soleus muscle in rats from the treadmill running. With long-term running, muscle contraction increases the number and percentage of muscle fibers, as well as muscle mass. This is in accordance with the results of a previous study, in which the effects of treadmill training on the soleus muscles were explored in rats after complete spinal cord transection at T8–T9. It was found that 9 weeks of treadmill running increased soleus muscle mass and cross-sectional area of muscle fibers in rats [51].

There were some limitations in the present study. Firstly, findings obtained from 5-month-old male SD rats may not be applicable to female rats. A recent study showed that loading of a 1-month-old female rat with a weight-bearing and moderate-intensity treadmill can promote bone formation and improve trabecular microstructure [19]. Future work will include the effects of treadmill exercise with different intensities on bone quality of female SD rats with the same age to obtain more comprehensive results and conclusions. Secondly, three exercise groups with different intensities (EX12, EX16 and EX20 groups) were used, and the total distance per day for each group was not equal, which may possibly affect the changes in bone quality. However, the exercise time for different groups was kept the same. Thus, it is meaningful to study the effects of different exercise intensities on bone quality. Future work can specify the total distance traveled and explore the effects of treadmill running on the bone of the small mammalian. Finally, the effects of different exercise intensities on nearly one single intact bone (femur) were investigated, which may not be applicable to other bones. However, considering that femur is load-bearing bone prone to fracture, this study is still meaningful.

## Conclusions

The results of this study demonstrated that after 4 weeks of treadmill running, among the three exercise intensities, the speed of 12 m/min could significantly affect the microstructure and mechanical properties of bone and improve the ultimate load of the soleus muscle. The treadmill running established in this study is effective and provides a reference for the selection of proper exercise intensity levels for adult males.

## Methods

This study was approved by the Medical Ethics Committee of the First Hospital of Jilin University (No. 2018-238). This study was in strict accordance with the requirement of the Laboratory Animal Standardization Committee. And all efforts were made to alleviate suffering of animals.

### Animals

Sixty 5-month-old male Sprague–Dawley rats were procured from the Animal Experimental Center of Jilin University. Animals were housed in groups of 4 (57 × 39 × 20 cm^3^), in laboratory cages under controlled laboratory conditions, a temperature of 23 ± 2 °C, and a relative humidity of 55 ± 5%, under a 12-h dark/light cycle. Rats were given freedom of movement in their cages, and provided ad libitum access to standard rodent food pellets (autoclaved diet National Institutes of Health-31 with 6% fat; 18% protein; Ca:P = 1:1; and supplemented vitamins and fortified minerals) and tap water. All the rats were healthy during the experimental period.

### Experimental design

**Fig. 3:**
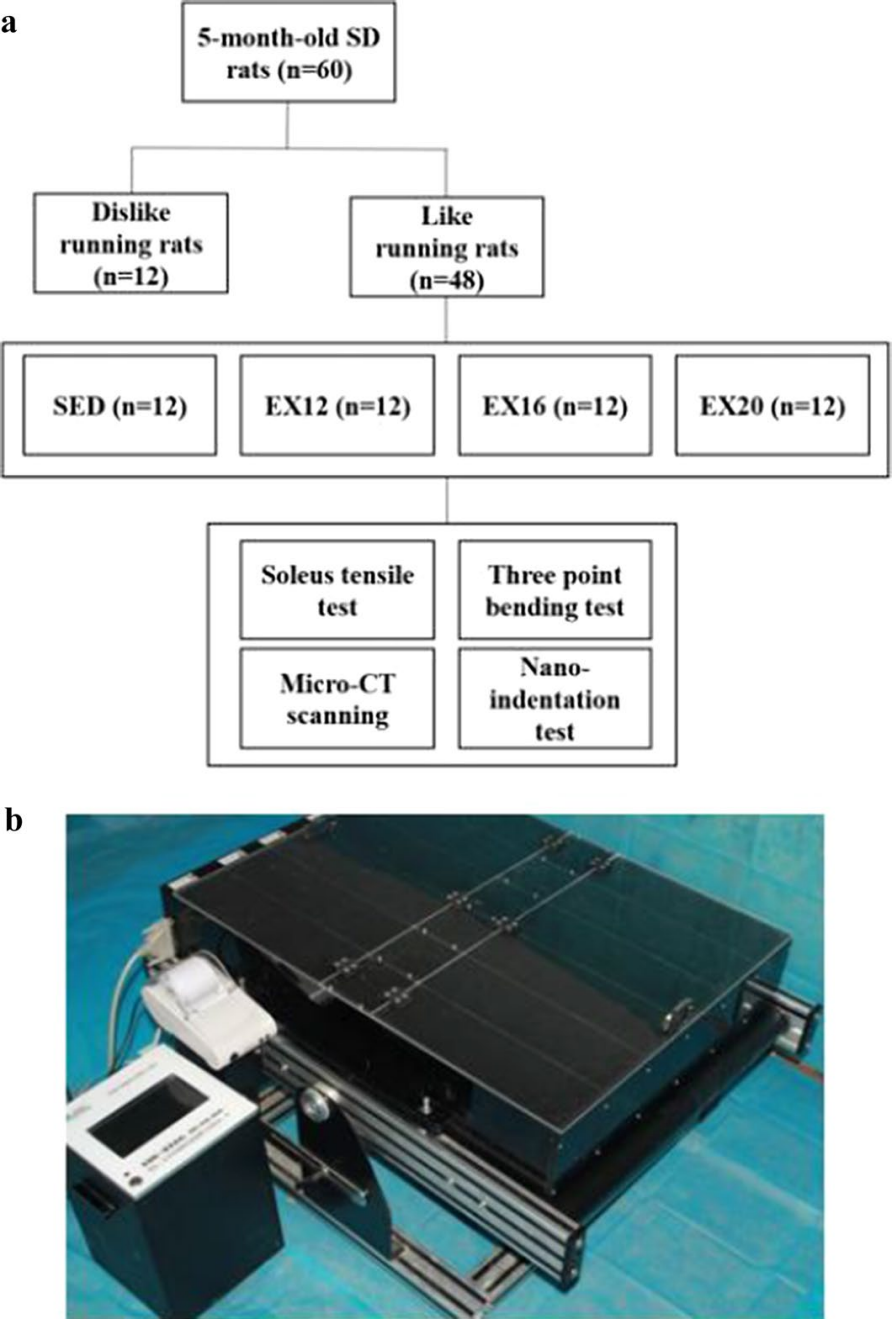
Temporal schematic of experiment and equipment for treadmill exercise. **a** The temporal schematic. **b** The equipment for treadmill exercise, which was composed of a treadmill platform and a controller

The temporal schematic is shown in Fig. 3a. The exercise groups underwent 1 week of adaption treadmill running. During the adaptive period, the rats were subjected to run on a flat treadmill (Fig. 3b) by gradually increasing the running speed from 10 m/min to 16 m/min, and 30 min/day, five times a week. In this stage, the rats were acclimated to treadmill running and those animals refused to run were eliminated. 48 rats with similar motor ability were selected and randomly assigned to sedentary control group (SED, *n* = 12) and exercise group (EX, *n* = 36). The rats in the exercise group were then randomly divided into 3 groups based on the average exercise speed: (1) Exercise group with a speed of 12 m/min (EX12); (2) Exercise group with a speed of 16 m/min (EX16); (3) Exercise group with a speed of 20 m/min (EX20). A 5-min “warming-up” at a speed of 10 m/min prior to each formal running was performed. Throughout the experimental period, the rats in the exercise groups ran on the treadmill for 30 min/day, 5 days a week, for 4 weeks. The rats in the sedentary control group were allowed to move freely in the cages, and were evaluated every day to ensure their health.

### Specimens preparation

48 h after the final exercise session, the rats were anesthetized with isoflurane, and then the blood sample with no less than 5 ml was obtained through the abdominal aorta of each rat. Serum was separated by centrifugation at 3000 rpm/min for 15 min. The obtained serum samples were then immediately stored in a refrigerator at − 20 °C until further analysis.

**Fig. 4:**
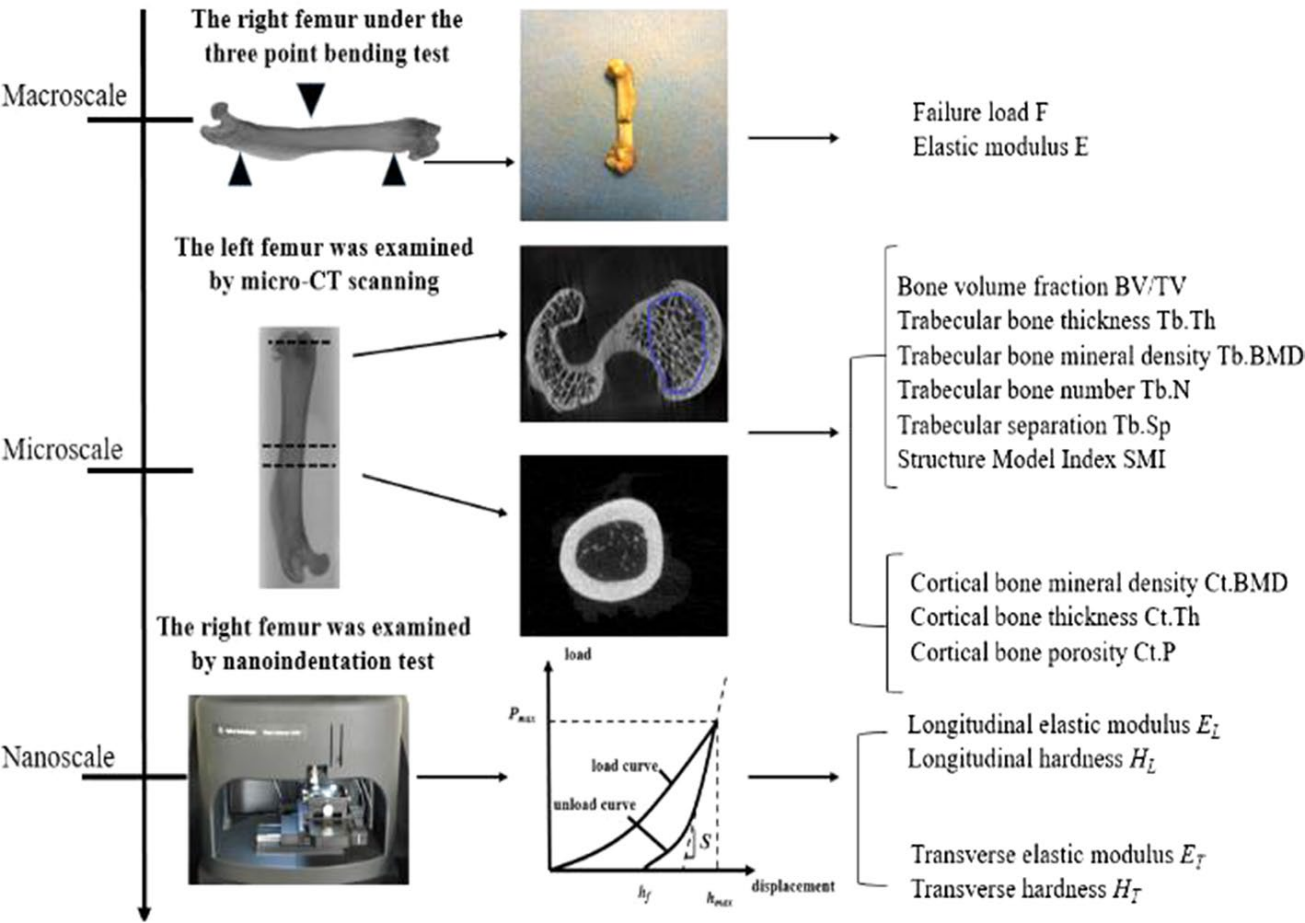
The multi-scale test of the femurs

The animals were then euthanized by cutting the abdominal aorta. The skin, muscles and tendons were peeled out carefully from both sides of the femur. The integrity of the left tibiofibula and muscles was maintained. All the dissected samples were wrapped in parafilm with saline solution and stored at − 20 °C until further analysis. Figure 4 represented the multi-scale test of the femur. For all the tests, the surveyors had no prior knowledge of the grouping of specimens.

### Soleus tensile test

**Fig. 5:**
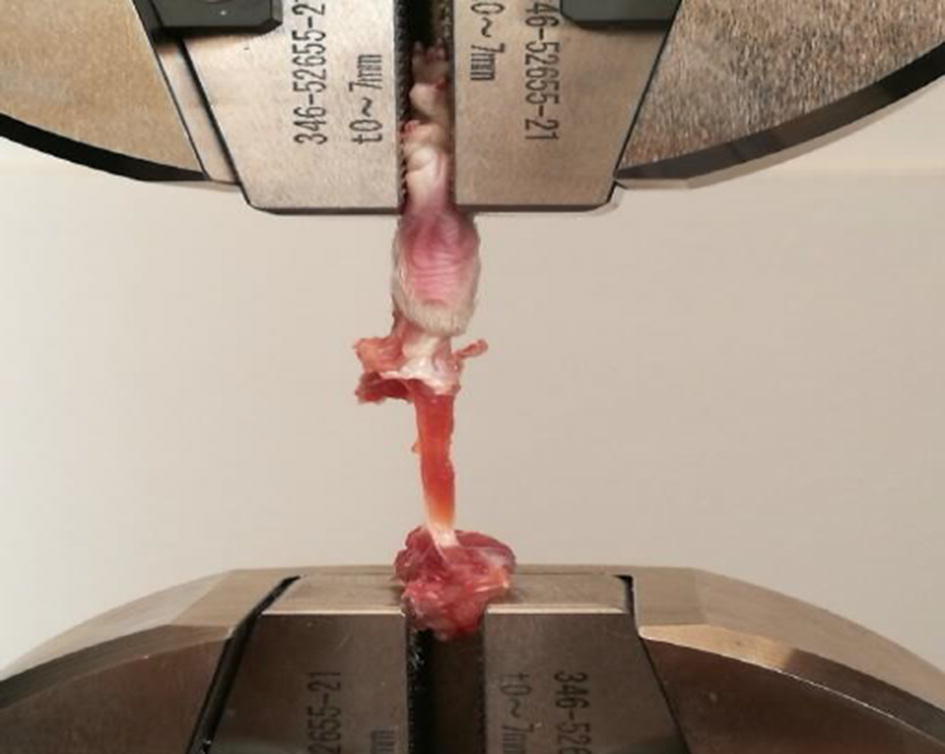
Soleus tensile test

The left tibia was thawed at room temperature for 6 h. The skin, surface muscle and gastrocnemius muscle at the back of the leg were cut away to expose the soleus muscle. During the sample preparation, the tibiofibula was dissected from the middle of the leg with the retained connection between the tendon and bone. This procedure was performed to ensure that the clamp can hold the sample appropriately, avoid direct contact with the muscle, and prevent stress concentration at the clamped position. The muscle specimen was prepared within 5 min, during which saline solution was titrated every 30 s to keep the sample moist. Immediately after the sample preparation, a tensile test was conducted using an electronic universal testing machine (AG-X plus, Shimadzu, Kyoto, Japan) (Fig. 5). The sample was loaded at a speed of 2 mm/min until the muscle fracture, and the load–displacement curve was recorded during the test. During the tensile phase, the soleus muscle was kept under moisture with dripping saline every 30 s. There were a total of 48 samples in this experiment, of which 46 samples were fractured at the position of muscle abdomen during the tensile test. One sample was injured by an operation error during peeling, and another sample was broken at the tendon position. These two samples were not counted in the final results. Immediately after muscle fracture, the soleus muscle was removed from the clamp and weighed on an electronic analytical balance. The intermediate processes were completed within 2 min.

### Serum analysis

Alkaline phosphatase (ALP) and tartrate-resistant acid phosphatase (TRACP) were quantitatively analyzed by enzyme-linked immunosorbent assay (enzyme-linked immunosorbent assay assay kit, NanJing, China) using ELIASA (CLARIOstar, BMG LABTECH, Germany), and bone formation and absorption rates were measured.

### Three-point bending mechanical test

**Fig. 6:**
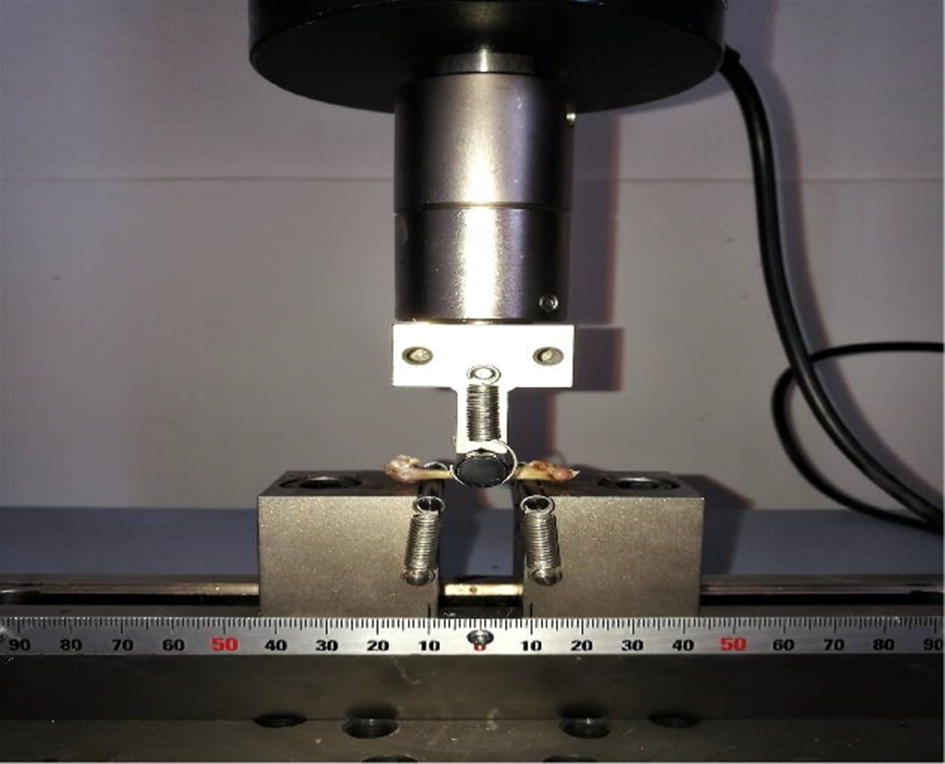
A right femur under the three-point bending mechanical test

Next, the right femur was thawed at room temperature and washed with saline solution to keep moist. As shown in Fig. 6, a three-point bending mechanical test was performed at room temperature of 22 °C using an electronic universal testing machine (AG-X plus, Shimadzu, Kyoto, Japan). The span of the fulcrum was adjusted to 20 mm, and the speed of the actuator was set at 1 mm/min. All samples were loaded in the same position with the middle of the femoral shaft as the loading point. The load–displacement curve was obtained with the bundled software (TRAPEZIUMX, Shimadzu, Kyoto, Japan), and the failure load was recorded. In this study, an elliptic ring was synthesized from a cross section of bone. The fractured samples were measured by a vernier caliper with the precision of 0.02 mm, and two surveyors who did not know the group information of the specimens were selected for testing. Each sample was measured for three times by each surveyor. The moment of inertia (*I*) and elastic modulus (*Em*) of the cross section were calculated using the following equations [21]:

1 $$I = \frac{{\pi \left( {BH^{3} - bh^{3} } \right)}}{64},$$

2 $$E_{m} = \frac{{L^{3} }}{48I}\left( {\frac{\Delta F}{\Delta f}} \right),$$

where *B* and *H* are the long and short axes of the outer ellipse of the cross section; *b* and *h* are the long and short axes of the inner ellipse of the cross section; *L* is the span of the two support point, and $\left( {\frac{\Delta F}{\Delta f}} \right)$ is the slope of the load–displacement curve.

### Micro-CT scanning

Left femurs were initially fixed with 80% ethanol (EtOH). Subsequently, femur samples were scanned using a benchtop micro-CT system (Skyscan 1076, Bruker-MicroCT, Belgium) at 18-μm voxel image resolution with 70 kV, 100 μA, and a 1.0-mm aluminum filter, to obtain three-dimensional (3D) microstructure parameters of trabecular bone and cortical bone. The projection data were then reconstructed with NRecon (Skyscan, Belgium) to create 3D images. The region of interest (ROI) was manually selected for the analysis of micro-CT images. The entire trabecular bone of the femoral lateral condyle was selected as trabecular ROI, and 5-mm cortical bone from femoral shaft to the proximal was selected as cortical ROI. Image analysis was performed with CTAn analysis software (CTAn, Skyscan, Belgium). The 3D morphometrical parameters of femoral head trabecular bone were measured, including bone mineral density (BMD), bone volume fraction (BV/TV), trabecular thickness (Tb.Th), trabecular number (Tb.N) and trabecular separation (Tb.Sp). The 3D microstructure parameters of femoral shaft cortical bone were also measured, including cortical bone mineral density (Ct.BMD), cortical bone thickness (Ct.Th) and cortical bone porosity (Ct.P).

### Nanoindentation test

**Fig. 7:**
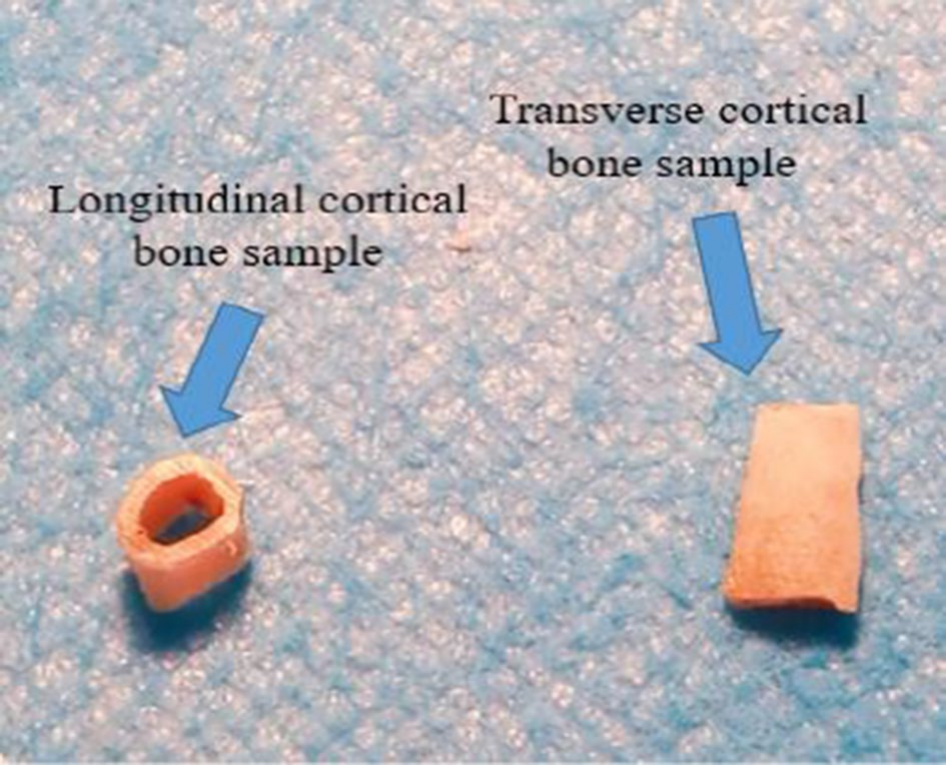
Preliminary preparation of the transverse and longitudinal samples of cortical bone. The sample on the left is a longitudinal cortical bone sample, and that on the right is a transverse cortical bone sample

Following the three-point bending test, all right femur samples were cleaned with deionized water. Two longitudinal cortical bone samples with equal size were cut along the axis of the femoral shaft. One of the samples was dissected through the medullary cavity along the axis of the femoral shaft to obtain the transverse cortical bone sample, and the other was used as the longitudinal cortical bone sample (Fig. 7). The specimens were dehydrated by a serial gradation ethyl alcohol (70%, 80%, 90%, and 100%) for 24–48 h per stage. After dehydration, all specimens were embedded in epoxy resin at room temperature [22]. All the embedded samples were metallographically polished using silicon carbide papers of decreasing grit sizes (600, 800, 1500, and 2000 grit), and subsequently on the microcloths with 0.05-µm grit of diamond suspension to obtain the smooth surfaces required for nanoindentation test. Finally, the samples were washed with deionized water to remove debris.

A Berkovich diamond indenter was used for the measurements. Nanoindentation tests were performed with the indenter speed of 750 μN/s and the indentation depth of 1000 nm, holding this load for 10 s, and finally unloading to 15% of the peak load at a rate equal to half that used during loading [23]. *E* and *H* were calculated using the method described by Oliver and Pharr [24].

*H* is calculated as the peak load (*P*_max_) divided by the projected contact area of the Berkovich tip (*A*):

3 $$H = \frac{{P_{ \text{max} } }}{A}.$$

*E*ef is the effective indentation modulus, and *S* is the linear slope of the unloading curve, and their relationship is:

4 $$S = \frac{2}{\sqrt \pi }\beta E_{\text{ef}} \sqrt A .$$

The *Eb* (*E*) is calculated as:

5 $$\frac{1}{{E_{\text{ef}} }} = \frac{{1 - v_{b}^{2} }}{{E_{b} }} + \frac{{1 - v_{i}^{2} }}{{E_{i} }},$$

where *ν* is the Poisson’s ratio, and *E* is the elastic modulus. The subscripts *b* and *i* refer to bone sample and the indenter, respectively. For the Berkovich indenter, *vi* = 0.07, *Ei* = 1140 GPa, *β *= 1.034. For the bone indenter, *νb* = 0.3.

### Statistical analysis

All statistical analyses were performed using SPSS 19.0 software. The mean value of each parameter of each group was calculated. All data obtained were analyzed using one-way analysis of variance (ANOVA) for differences among all groups. If significant difference was observed, the least significant difference test was used for post hoc comparison to compare the difference between every two groups. The significance level of *p* was chosen to be 0.05.

## Data Availability

The datasets used and/or analyzed during the current study are available from the corresponding author on reasonable request.

## Abbreviations

ALP: alkaline phosphatase
TRACP: tartrate-resistant acid phosphatase
ROI: region of interest
BMD: bone mineral density
BV/TV: bone volume fraction
Tb.Th: trabecular thickness
Tb.N: trabecular number
Tb.Sp: trabecular separation
SMI: structure model index
Ct.BMD: cortical bone density
Ct.Th: cortical bone thickness
Ct.P: cortical bone porosity
Ct.L: longitudinal cortical bone
Ct.T: transverse cortical bone
*E*: modulus of indentation
*H*: indentation hardness
*E/H*: ratio of indentation modulus to indentation hardness
*EL*: longitudinal modulus of indentation
*ET*: transverse modulus of indentation
*HL*: longitudinal indentation hardness
*HT*: transverse indentation hardness
*EL/HL*: ratio of longitudinal indentation modulus to indentation hardness
*ET/HT*: ratio of transverse indentation modulus to indentation hardness
